# Long-term persistence of pneumococcal antibodies 5 years after a sequential PCV13 and PPSV23 vaccination in kidney transplant recipients: indications for revaccination

**DOI:** 10.1128/msphere.00820-25

**Published:** 2026-02-26

**Authors:** Lukas van de Sand, Monika Lindemann, Kim L. Völk, Sebastian Dolff, Oliver Witzke, Adalbert Krawczyk, Benjamin Wilde, Nils Mülling

**Affiliations:** 1 Department of Infectious Diseases and Nephrology, University Hospital Essen, University Duisburg-Essen 536544, Essen, Germany; 2 Institute for Transfusion Medicine, University Hospital Essen, University Duisburg-Essen 549321, Essen, Germany; 3 Institute for Virology, University Medicine Essen, University of Duisburg-Essen 27170, Essen, Germany; Universitat Munster Institut fur Zellulare Virologie, Münster, Germany

**Keywords:** *Streptococcus pneumoniae*, vaccination, kidney transplantation, sequential vaccination, serotype-specific immunity, pneumococcal antigens

## Abstract

Pneumococcal vaccination is essential to prevent invasive *Streptococcus pneumoniae* infections in immunocompromised individuals, including kidney transplant recipients. Current recommendations favor single-dose immunization with higher-valent conjugate vaccines, including PCV20 or PCV21. Five years ago, sequential administration of the 13-valent conjugate vaccine (PCV13) followed by the 23-valent polysaccharide vaccine (PPSV23) represented standard of care. Long-term data on antibody persistence after this regimen remain limited. In this prospective 5-year follow-up study, 46 kidney transplant recipients previously vaccinated sequentially with PCV13 and PPSV23 were re-evaluated. Nine patients were lost to follow-up, and 11 had died during the observation period. The remaining 26 participants were re-enrolled and completed the 5-year assessment. Global and serotype-specific IgG anti-pneumococcal antibody concentrations were quantified and compared with baseline and 12-month post-vaccination levels. Clinical outcomes, including pneumococcal infections and allograft status, were recorded. Five years after vaccination, antibody concentrations remained above baseline levels in most participants. Mean IgG levels were still approximately threefold higher than pre-vaccination values. Even for serotype-specific responses, mean antibody levels showed minimal changes compared with those measured 12 months after the first vaccination, although absolute titers remained considerably lower than those observed in healthy individuals. No cases of pneumococcal pneumonia or vaccine-associated allograft rejection occurred during follow-up. Sequential vaccination elicits durable immune responses in kidney transplant recipients, persisting up to 5 years post-immunization. With the availability of new vaccines covering additional serotypes, and given the generally lower antibody responses in this high-risk population, a booster with PCV20 or PCV21 appears advisable to enhance and broaden protection.

**IMPORTANCE:**

Kidney transplant recipients are at high risk for invasive pneumococcal disease, yet long-term vaccine-induced immunity in this population remains poorly defined. This study provides one of the longest longitudinal assessments of humoral responses following sequential PCV13 and PPSV23 vaccination, extending to 5 years post-immunization. We demonstrate sustained but heterogeneous antibody persistence and serotype-dependent responses to PCV20 booster vaccination. These results are directly relevant to transplant clinicians, vaccinologists, and public health policy, offering critical insight into long-term pneumococcal immunity in immunocompromised hosts and guiding future vaccine scheduling in solid organ transplantation.

## INTRODUCTION

Pneumococcal infections remain one of the main causes of morbidity and mortality in kidney transplant recipients (KTRs). Ongoing immunosuppression makes these patients particularly vulnerable. Pulmonary infections, including pneumonia, are a leading cause of morbidity and mortality in organ transplant recipients (1). KTRs are also at an elevated risk for invasive pneumococcal disease (IPD). Various studies indicate that the incidence of IPD in this population is significantly higher than in the general population (2, 3).

Vaccination represents a key preventive measure against pneumococcal disease. Until 2024, the German Standing Committee on Vaccination (STIKO) recommended a sequential vaccination approach for immunocompromised individuals, including KTRs (4). This vaccination schedule consisted of an initial dose of the 13-valent pneumococcal conjugate vaccine (PCV13), followed by a dose of the 23-valent pneumococcal polysaccharide vaccine (PPSV23) 6–12 months later. However, with the introduction of the 20-valent pneumococcal conjugate vaccine (PCV20), marketed as Prevenar 20, current guidelines now recommend its use in this high-risk group. The STIKO has updated its recommendations to include PCV20 for adults aged 18 years and older with congenital or acquired immunodeficiencies, including those undergoing immunosuppressive therapy such as kidney transplantation (4).

The effectiveness of pneumococcal vaccination in KTRs has been demonstrated in several studies, which have shown that immunization can lead to increased antibody concentrations and may reduce the incidence of pneumococcal infections (5–7). KTRs who received sequential vaccination with PCV13 and PPSV23 exhibited significant rises in serotype-specific and overall anti-pneumococcal antibody concentrations (5, 6). However, despite these increases, antibody levels in KTRs remained lower than those observed in healthy individuals (5–7). The magnitude of the antibody response also depended on the type of immunosuppressive therapy: patients not receiving mycophenolic acid exhibited significantly higher global IgG and IgG2 antibody levels at all time points after vaccination (6).

Despite these recommendations, long-term data on the persistence of pneumococcal antibodies in KTRs remain limited. In healthy older adult cohorts without major comorbidities, some data are available: Among 62 individuals aged 65–88 years who received the 23-valent polysaccharide vaccine, geometric mean antibody concentrations (GMCs) measured at 3 years had declined to levels similar to pre-vaccination for several serotypes (e.g., types 6B and 19F) and, for other serotypes (4, 9V, and 23F), even close to the baseline levels (8). In another study of elderly adults aged 60–67 years, antibody levels were significantly higher four weeks after vaccination in most participants. According to established revaccination guidelines, 60% of these individuals required revaccination 5 years after the primary dose, while the remaining 40% would need reassessment at a later time point (9). Similarly, long-term follow-up data demonstrated that antibody concentrations against pneumococcal capsular polysaccharides persisted for several years but gradually declined, although revaccination effectively restored antibody levels (10).

Comparable long-term immunogenicity data in immunosuppressed populations are scarce. In a recent nested case-control analysis among adults with immune-mediated inflammatory diseases, pneumococcal vaccination was, however, associated with a 20–40% lower risk of three outcomes: lower respiratory tract infection treated with outpatient antibiotics, hospitalization for pneumonia, and death from pneumonia (11). These findings underscore the protective clinical effect of pneumococcal vaccination in immunocompromised individuals but highlight the lack of detailed evidence on long-term antibody persistence and efficiency in KTRs.

## MATERIALS AND METHODS

### Study population

This follow-up study was conducted at the University Hospital Essen to evaluate the long-term persistence of pneumococcal antibodies in kidney transplant recipients (KTRs) who had previously participated in a prospective vaccination trial investigating the immunogenicity of sequential pneumococcal vaccination with the 13-valent conjugate vaccine (PCV13) followed by the 23-valent polysaccharide vaccine (PPSV23) (6). At that time, national vaccination guidelines in Germany recommended a sequential vaccination schedule with PCV13 and PPSV23 for immunocompromised patients, including solid organ transplant recipients (12).

In the original study, 46 clinically stable adult KTRs were sequentially immunized with PCV13 and PPSV23 six months apart. In the present study, patients were re-evaluated, using previously published antibody concentrations up to month 12 (6) and adding newly collected data at 60 months for long-term assessment. Of the 46 initially enrolled recipients, 26 (57%) were successfully re-included in the present analysis. Eleven participants (24%) had died during the follow-up period, and nine (19%) were lost to follow-up due to return to dialysis treatment or inability to establish contact. During the 5-year interval between the initial study and follow-up, four participants received an additional pneumococcal vaccination outside of the study protocol. No cases of pneumococcal pneumonia were reported among the 26 re-evaluated patients. Demographic and clinical characteristics were extracted from medical records at the time of follow-up sampling. Basic patient information is provided in Table 1.

**TABLE 1:** Basic patient characteristics

Parameter	Median (range) or number (no.)	
Median age (range), years^*a*^	60 (28–83)	
Gender (male/female)	17/9	
Median interval from transplantation to vaccination (range), months^*a*^	34 (3–203)	
Median serum creatinine (range), mg/dL		
Pre-vaccination	1.40 (0.96–3.55)	
Month 12 post-vaccination	1.38 (0.97–3.85)	
Month 60 post-vaccination	1.48 (0.83–4.31)	
Immunosuppression, no.	At baseline	At month 60
Cyclosporine A	2 (8%)	2 (8%)
Tacrolimus	18 (69%)	18 (69%)
Mycophenolic acid	20 (77%)	18 (69%)
mTOR inhibitors	4 (15%)	5 (19%)
Corticosteroids	22 (85%)	25 (96%)
Belatacept	4 (15%)	4 (15%)
Kidney transplantation, no.		
First	23	
Second	3	
Ever received additional pneumococcal vaccine after recruitment	4	

^*a*^ At the time of first blood sampling; mTOR = mammalian target of rapamycin.

### Vaccination history

Participants in the original study received one intramuscular dose of PCV13, containing polysaccharides from serotypes 1, 3, 4, 5, 6A, 6B, 7F, 9V, 14, 18C, 19F, 19A, and 23F, each conjugated to a non-toxic diphtheria CRM197 carrier protein (13). Six months later, a single intramuscular dose of PPSV23 was administered, containing 25 µg of each of the following serotypes: 1, 2, 3, 4, 5, 6B, 7F, 8, 9N, 9V, 10A, 11A, 12F, 14, 15B, 17F, 18C, 19F, 19A, 20, 22F, 23F, and 33F (14). Both vaccines were injected into the deltoid muscle.

For the 5-year follow-up, vaccination records of all participants were reviewed. Four patients received an additional pneumococcal vaccination during the observation period, which was documented and accounted for in the analysis. Specifically, these patients received Prevenar 20 between January 2024 and February 2025.

### Blood sampling and serological analysis

For the current study, blood samples were collected approximately 5 years after completion of the sequential vaccination. Serum was separated and stored at −80°C until analysis.

Serotype-specific IgG antibody concentrations against pneumococci were determined by enzyme-linked immunosorbent assay (ELISA) according to the World Health Organization (WHO) reference protocol as previously described, using 96-well microplates coated with pneumococcal polysaccharides (serotypes 2, 3, 6A, 9N, 11A, and 14) (15). The reference and quality control serum 007sp, kindly provided by Mustafa Akkoyunlu (Pneumococcal Reference Laboratory, Birmingham, AL, USA), was used for quantification. Antibody concentrations were expressed in mg/L, with a lower limit of detection of 0.01 mg/L.

Additionally, total pneumococcal IgG, IgG2, IgA, and IgM antibodies recognizing 23 serotypes were quantified using the commercial VaccZyme Pneumococcal Capsular Polysaccharide Assay (The Binding Site, Birmingham, UK), according to the manufacturer’s instructions. All assays were performed in duplicate. Optical density values were measured at 450 nm using a Tristar 3 multimode ELISA plate reader (Berthold Technologies, Bad Wildbad, Germany).

Serum antibody concentrations were calculated using GraphPad Prism version 10.6.1 (GraphPad Software, San Diego, CA, USA) by log-linear regression analysis.

### Statistical analysis

Statistical evaluation was performed using GraphPad Prism (version 10.6.1, GraphPad Software). Continuous variables were summarized as medians and ranges. Data distribution was assessed by the Shapiro-Wilk test. Non-parametric tests (Wilcoxon signed-rank test and Mann-Whitney *U* test) were used for paired and unpaired comparisons, respectively.

Changes in antibody concentrations between the time points were analyzed using the Kruskal-Wallis test, with Dunn’s correction for multiple comparisons. A two-sided *P* value < 0.05 was considered statistically significant.

## RESULTS

### Clinical course of the study cohort

**Fig 1:**
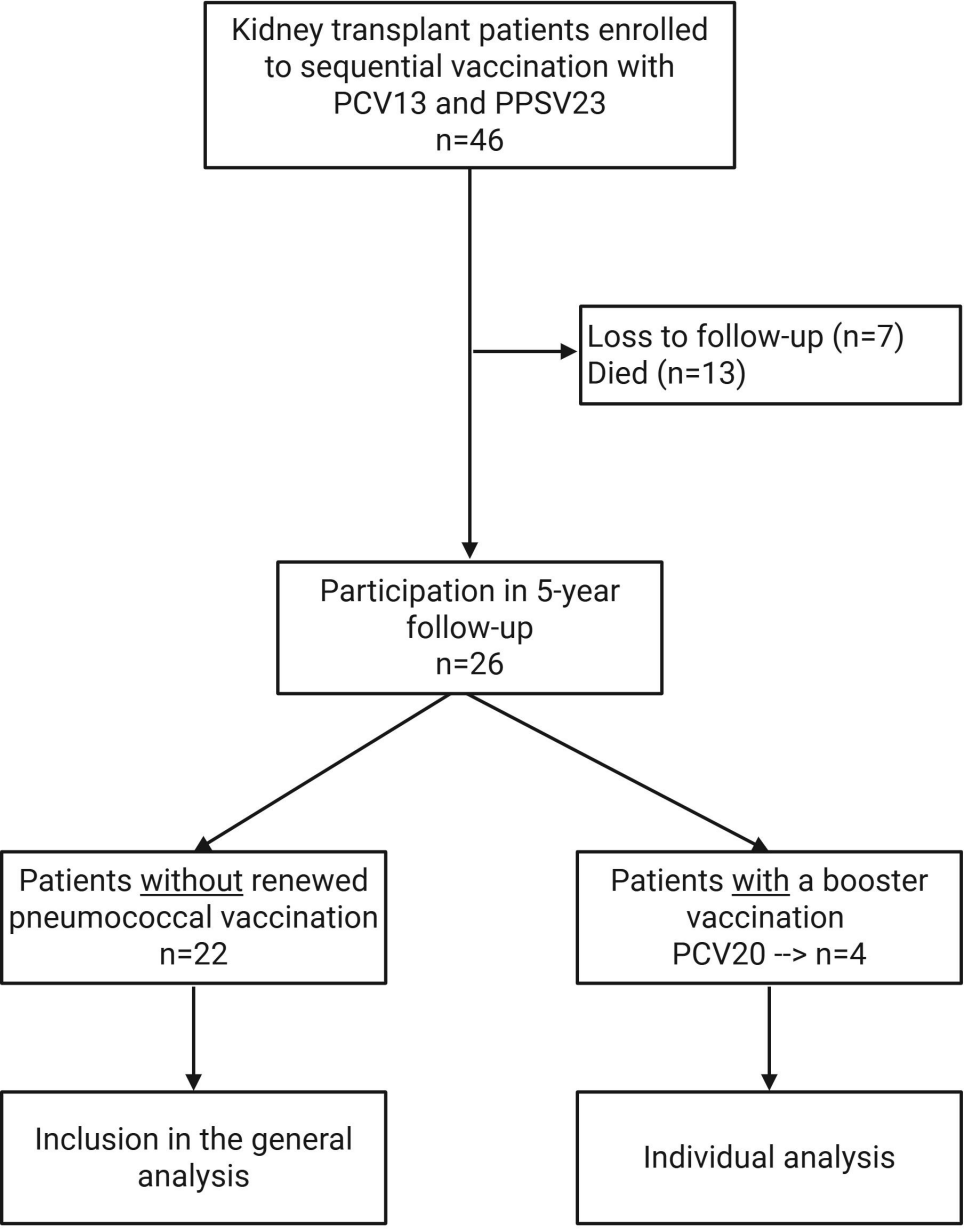
Flow diagram of the study cohort.

A total of 46 clinically stable adult kidney transplant recipients (KTRs) were included in the original study and received sequential pneumococcal vaccination with PCV13 followed by PPSV23 at a 6-month interval. Approximately 5 years after completion of the initial immunization schedule, all surviving participants were invited to undergo serological reassessment. Of the original 46 participants, 26 individuals were successfully re-enrolled in the present follow-up analysis. Eleven patients had died during the observation period, while nine could not be re-evaluated due to return to dialysis or loss of contact. In addition, four participants had received an additional pneumococcal vaccination with PCV20 during the follow-up period. These individuals were analyzed separately in the following evaluation. A flowchart of patient inclusion and follow-up is presented in Fig. 1.

Among the deceased participants, detailed information on the cause of death was available for four individuals, while data were missing for seven cases. Reported causes of death included metastatic urothelial carcinoma of the transplant kidney (*n* = 1), cardiogenic shock following non-ST-elevation myocardial infarction (*n* = 1), COVID-19-associated acute respiratory distress syndrome (*n* = 1), and pneumogenic sepsis with a positive pneumococcal antigen test, accompanied by pericardial effusion and subsequent in-hospital cardiac arrest (*n* = 1). The latter represents the only documented case of pneumococcal infection within the entire original cohort of 46 patients. In addition, one patient developed chronic allograft rejection during the observation period.

### Serotype-specific pneumococcal antibodies over 5 years

**Fig 2:**
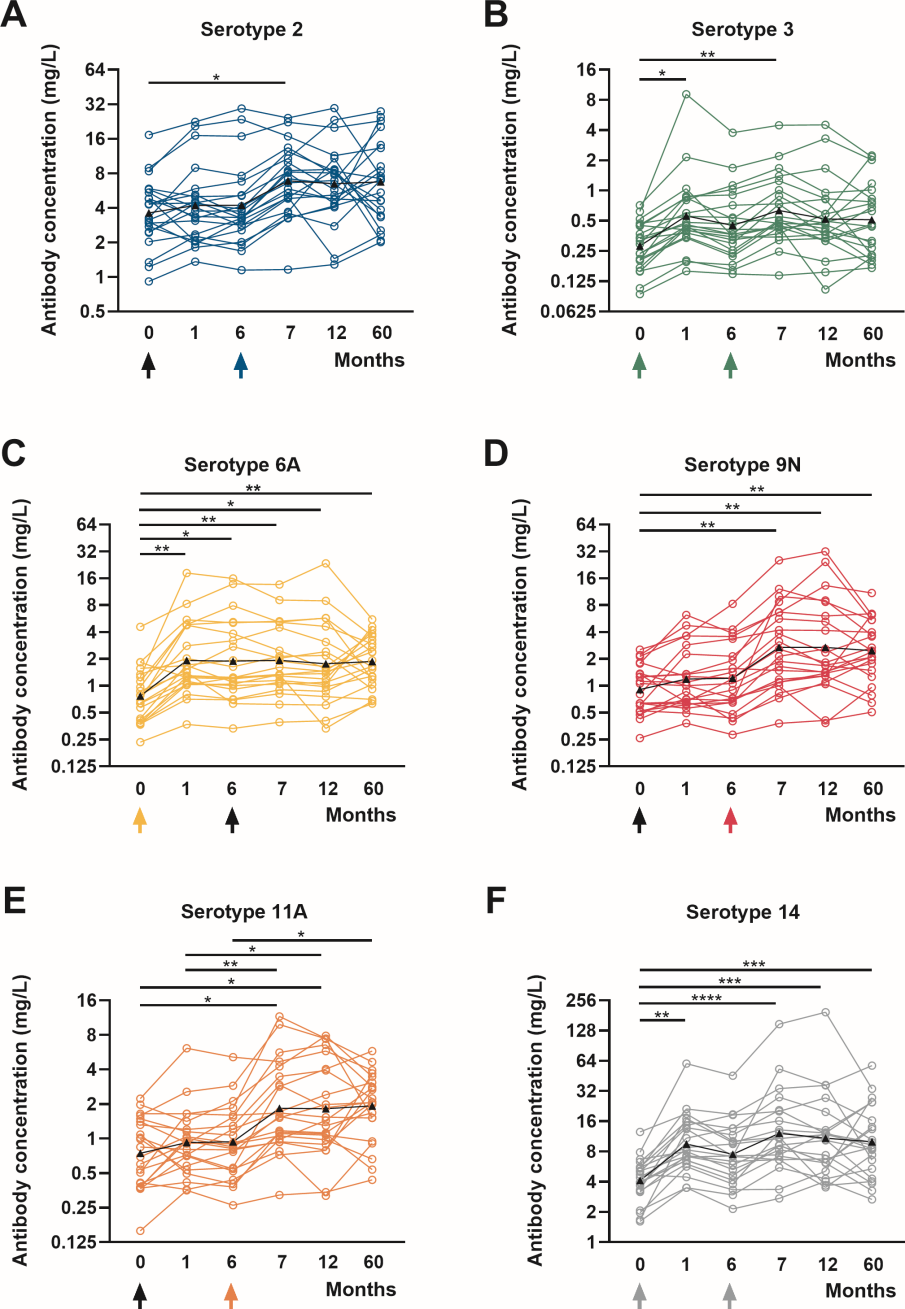
Serotype-specific pneumococcal antibody time courses prior to and after immunization with PCV13 (at month 0) and with PPSV23 (at month 6). Serotypes 2, 3, 6A, 9N, 11A, and 14 are displayed. The presence of the matching serotype in the vaccine is shown by colored arrows. Geometric mean values are shown by the triangle-adorned black line. The results are shown on a logarithmic scale (log2). The Kruskal-Wallis test and Dunn’s multiple comparisons test were used to examine data from 22 KTRs. *P < 0.05, **P < 0.01, ***P < 0.0005, and ****P < 0.0001.

**Fig 3:**
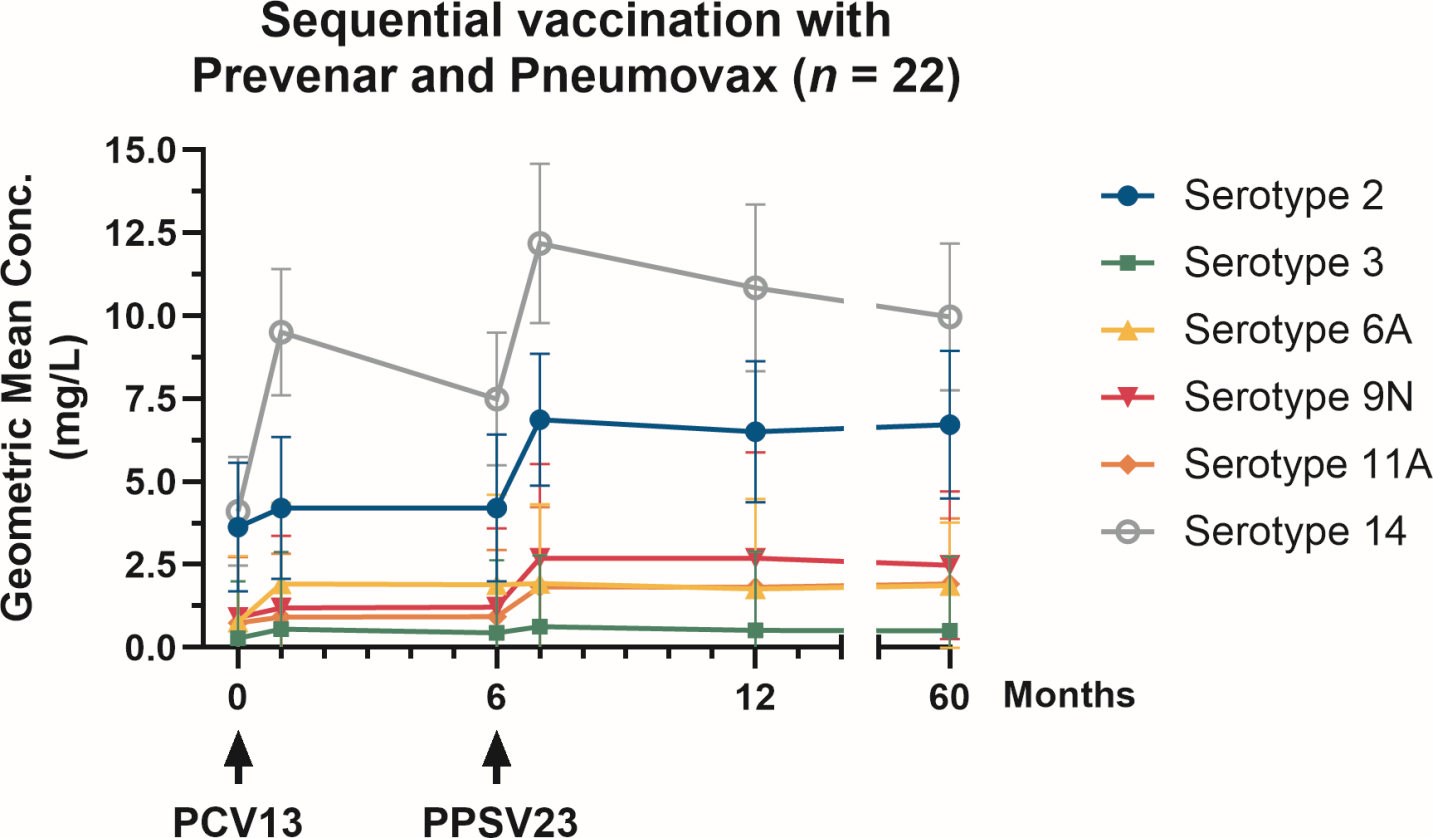
Pneumococcal antibody concentrations by serotype over a period of 5 years following first vaccination. For 22 kidney transplant recipients, antibodies were measured before their first PCV13 vaccine (M0), 1 month later (M1), before their second PPSV23 vaccination (M6), at month 7 (M7), at month 12 (M12), and 5 years later (M60). Pneumococcal antibodies are given as geometric mean concentration and geometric standard deviation factor. The time of vaccination is indicated by an arrow.

We re-enrolled 26 patients from the original study; here, we report results for the 22 patients who had not received an additional PCV20 booster. Serum samples collected over 5 years were analyzed using a standardized WHO pneumococcal ELISA for six serotypes: 2, 3, 6A, 9N, 11A, and 14 (Fig. 2). Geometric mean concentrations (GMCs) and interquartile range (IQR) are summarized in Table 2 and Fig. 3.

**TABLE 2:** Serologic responses to sequential pneumococcal vaccination

Time point	Geometric mean concentration (IQR), mg/L, *n* = 22, for serotype:					
	2	3	6A	9N	11A	14
Month 0	3.6 (2.5–5.4)	0.3 (0.2–0.5)	0.8 (0.4–1.3)	0.9 (0.5–1.4)	0.7 (0.4–1.4)	4.1 (3.3–5.6)
Month 1	4.2 (2.2–5.2)	0.6 (0.4–0.8)	1.9 (1.1–4.8)	1.2 (0.7–2.3)	0.9 (0.6–1.2)	9.5 (6.6–14.5)
Month 6	4.2 (2.8–5.2)	0.4 (0.3–0.7)	1.9 (1.0–3.8)	1.2 (0.7–2.1)	0.9 (0.5–1.5)	7.5 (4.6–11.7)
Month 7	6.9 (4.8–10.8)	0.6 (0.4–1.0)	1.9 (1.1–4.5)	2.7 (1.1–6.6)	1.8 (1.0–3.5)	12.2 (7.2–20.1)
Month 12	6.5 (4.4–8.7)	0.5 (0.3–0.8)	1.8 (1.0–3.1)	2.7 (1.3–6.1)	1.8 (1.0–4.0)	10.9 (6.2–13.1)
Month 60	6.7 (3.4–13.3)	0.5 (0.2–0.8)	1.9 (1.2–3.3)	2.5 (1.5–5.4)	1.9 (1.5–3.1)	10.0 (5.4–16.4)

Comparing pre-vaccination and month 60 antibody concentrations, a significant increase was still observed for serotypes 6A, 9N, and 14. The highest antibody levels were generally measured at month 7, 1 month after vaccination with PPSV23. Over the entire 5-year follow-up, no significant decline in antibody levels was observed for any serotype. GMCs were lowest for serotype 3 throughout the study. GMCs for serotype 3 increased from 0.3 mg/L pre-vaccination to 0.6 mg/L at month 1 (*P* < 0.05), remained at 0.6 mg/L at month 7 (*P* < 0.05), and then declined slightly to 0.5 mg/L at 5 years. In contrast, serotype 14, which is also targeted by both PCV13 and PPSV23, demonstrated a markedly stronger response. After the first vaccination, GMCs reached 9.5 mg/L and were further boosted to 12.2 mg/L following PPSV23, corresponding to a threefold increase compared with baseline. Five years later, antibody levels showed only a slight decline.

For serotype 2, which is included only in PPSV23, GMCs rose from 3.6 mg/L pre-vaccination to 6.9 mg/L at month 7 (*P* < 0.05). At months 12 and 60, no significant difference compared with baseline was observed, with only a very modest decrease to 6.7 mg/L at month 60. Serotype 9N also occurs only in PPSV23 and antibodies against this serotype showed a peak GMC of 2.7 mg/L after vaccination, with a slight decrease to 2.5 mg/L at 5 years, remaining significantly elevated compared with baseline (*P* < 0.01). Similarly, antibodies against serotype 11A showed a stable GMC of 1.9 mg/L at month 60 (*P* < 0.05). Serotype 6A, included only in PCV13, showed surprisingly stable antibody responses despite being a single-dose antigen in this study. GMCs remained at 1.9 mg/L both one month after vaccination and 5 years later, indicating sustained long-term persistence.

When antibody concentrations were evaluated against the American Academy of Allergy, Asthma, and Immunology (AAAAI)-defined protective threshold of 1.3 mg/L (16), clear serotype-specific differences emerged. Considering the AAAAI definition, 5 years after sequential vaccination, all patients maintained protective antibody levels for serotype 2, reflecting sustained long-term immunity (Table 3). In contrast, only 14% of patients retained protective concentrations for serotype 3, consistent with its constantly low GMCs over time. Additionally, we applied serotype-specific cut-off values, which had been established in a group of 100 healthy, pneumococcal infection-free, and pneumococcal vaccination-naïve adults, because the immunogenicity of each serotype is different (17). Notably, for serotypes 2 and 3, there was no difference when considering the general and serotype-specific cut-off values. Antibodies against serotype 6A showed moderate persistence, with 64% of patients remaining above the protective 1.3 mg/L threshold. For serotype 9N, 77% of patients maintained protective antibody levels, although only a small fraction exceeded the serotype-specific cut-off. Similarly, antibodies against serotype 11A remained above the threshold in 77% of patients, with 41% surpassing the serotype-specific value. Serotype 14 antibodies demonstrated the second strongest long-term response, with all patients retaining antibody concentrations above 1.3 mg/L and 68% exceeding the serotype-specific threshold, corresponding to a geometric mean fold increase (GMFI) of over 2.4. Only antibodies against serotypes 2 and 3 showed a GMFI below 2.

**TABLE 3:** Comparison of antibody concentrations relative to baseline (M0) 5 years after vaccination^*a*^

Serotype	Percentage cut-off (1.3 mg/L)	Percentage serotype-specific cut-off	Geometric mean fold increase
Serotype 2	100	100	1.9
Serotype 3	14	14	1.8
Serotype 6A	64	N/A^*b*^	2.4
Serotype 9N	77	5	2.7
Serotype 11A	77	41	2.6
Serotype 14	100	68	2.4

^*a*^ The table shows the percentage of patients who maintained antibody concentrations above the defined cut-off value 5 years after vaccination and the geometric mean fold increase (GMFI) during this period. For the purposes of evaluation, an adequate IgG antibody response to an individual serotype was arbitrarily defined as a postimmunization antibody concentration ≥1.3 mg/L (16). Serotype-specific reference concentrations were assumed as previously described: serotype 2 = 1.0 mg/L, serotype 3 = 1.8 mg/L, serotype 9N = 9.15 mg/L, serotype 11A = 2.4 mg/L, and serotype 14 = 7.0 mg/L (17).

^*b*^ N/A, not applicable due to lack of reference value (serotype 6A).

### Global immunoglobulin responses

**Fig 4:**
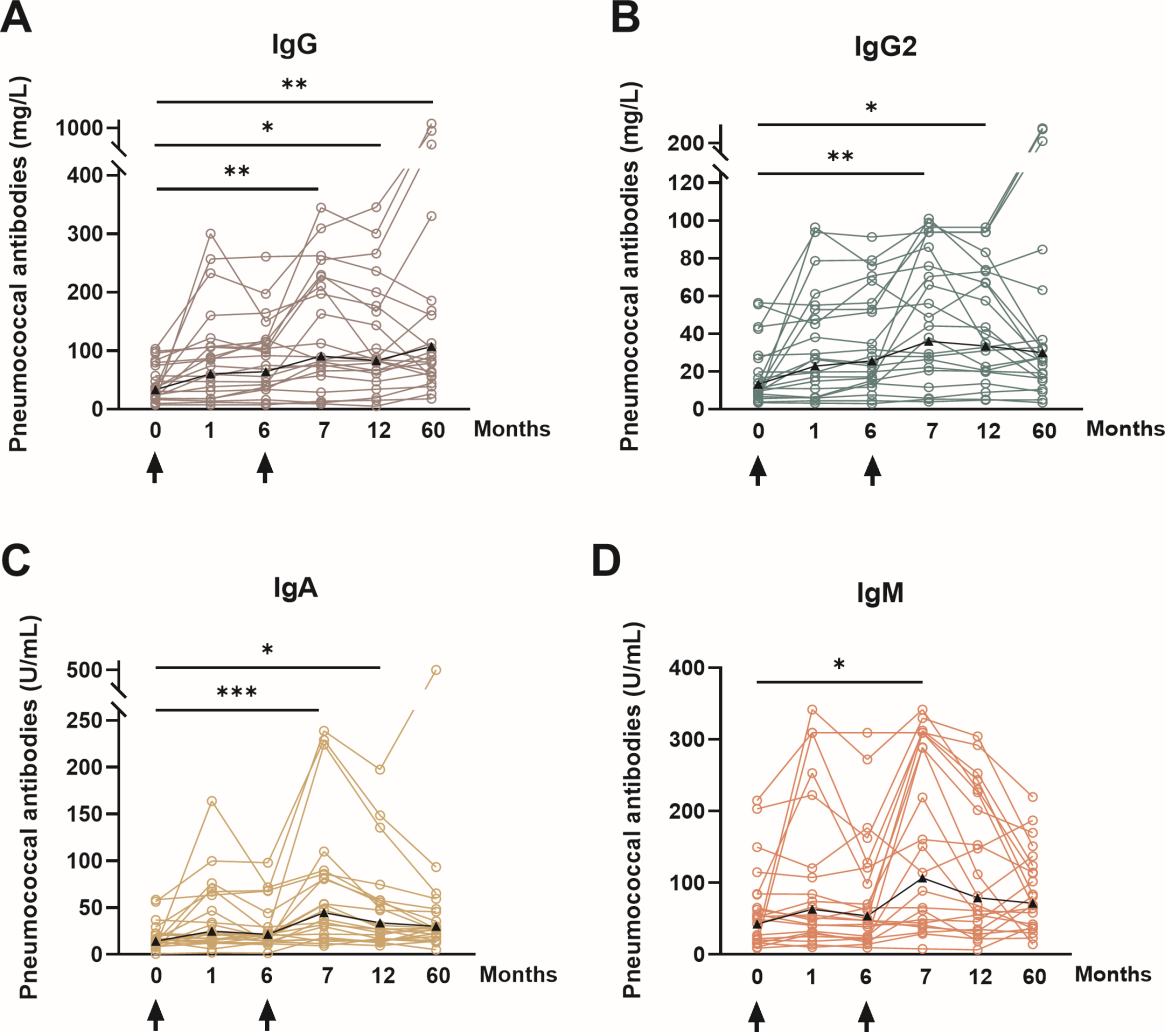
Global, individualized pneumococcal antibody courses after sequential vaccination. The time of vaccination is indicated by an arrow (PCV13 and subsequently PPSV23). Based on a commercially available ELISA that measures antibodies against 23 serotypes (global ELISA), (**A**) IgG antibodies, (**B**) IgG2, (**C**) IgA, and (**D**) IgM. The Kruskal-Wallis test and Dunn’s multiple comparisons test were used to examine data from 22 KTRs. *P < 0.05, **P < 0.01, and ***P < 0.0005.

**Fig 5:**
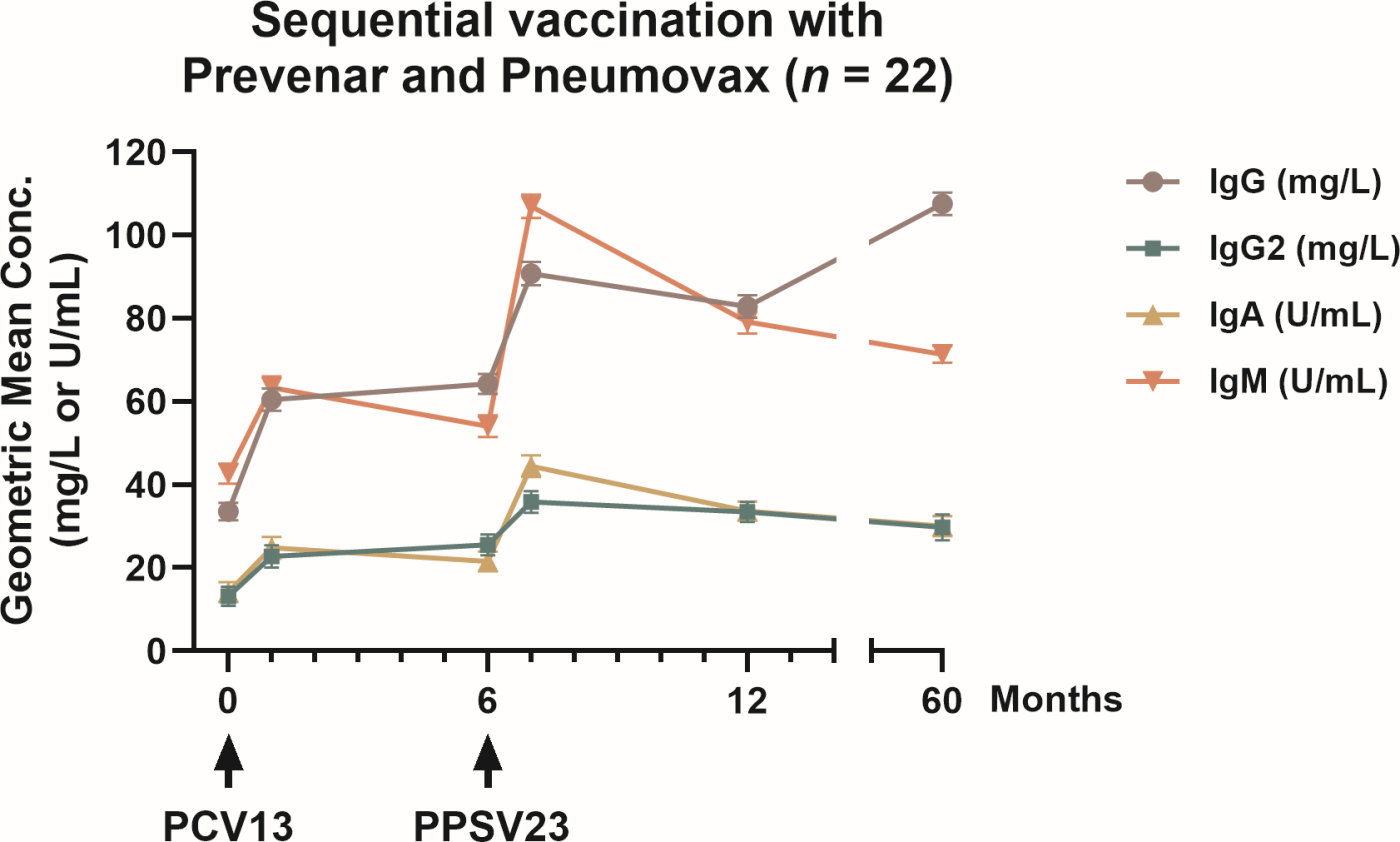
Time courses of global pneumococcal antibodies in kidney transplant patients. Data are from the present cohort of 22 patients who had sequential vaccinations with PCV13 (at month 0) and PPSV23 (at month 6). A commercially available ELISA, which measures antibodies against 23 serotypes (global ELISA), was used to determine IgG, IgG2, IgA, and IgM antibodies. The geometric mean concentration and geometric standard deviation factor are presented. Arrow indicates time of immunization.

Global IgG, IgG2, IgA, and IgM ELISAs were performed for 23 pneumococcal serotypes in 22 patients. The GMC of total IgG antibodies against all serotypes was 33.5 mg/L pre-vaccination, 90.7 mg/L at month 7, and 107.5 mg/L at month 60 (Fig. 4A and 5; Table 4). A significant increase in IgG antibody levels was observed at month 7 (*P* < 0.01), month 12 (*P* < 0.05), and month 60 (*P* < 0.01) compared with baseline, indicating a strong and durable humoral response over the 5-year observation period. Remarkably, higher GMC values were measured at month 60 than at month 12, which can most likely be attributed to three individual outliers with exceptionally high antibody concentrations. The course of IgG2 antibody concentrations closely followed that of total IgG, though at lower levels, as expected for this subclass (Fig. 4A and B, 5). The GMC of IgG2 increased from 13.2 mg/L pre-vaccination to 35.9 mg/L at month 7 (*P* < 0.01), reflecting a significant rise after the second vaccination. At month 12, antibody levels remained significantly higher than baseline (*P* < 0.05). However, at month 60, IgG2 concentrations had declined to 29.8 mg/L, which was no longer significantly different from baseline. IgA antibody concentrations showed a similar kinetic pattern with a clear boost effect after the second vaccination. GMCs increased from 14.1 mg/L pre-vaccination to 44.5 mg/L at month 7 (*P* < 0.0005), followed by a gradual decrease to 33.6 mg/L at month 12 and 30.1 mg/L at month 60. Finally, IgM responses demonstrated a pronounced but transient boost after PPSV23 administration. The GMC of IgM increased from 42.6 mg/L pre-vaccination to 106.9 mg/L at month 7 (*P* < 0.05), confirming a strong early polysaccharide-specific response. Subsequently, IgM levels declined to 79.2 mg/L at month 12 and 71.4 mg/L at month 60, remaining above pre-vaccination levels but without statistical significance compared with baseline.

**TABLE 4:** Pneumococcal antibody levels in 22 kidney transplant recipients who received the PCV13 vaccine at month 0 and the PPSV23 vaccine at month 6 were tracked throughout time, and their antibody concentrations were compared to those of a healthy reference group^*a*^

Time point	IgG		IgG2		IgA		IgM	
	GMC	PRC	GMC	PRC	GMC	PRC	GMC	PRC
Month 0	33.5	36	13.2	27	14.1	27	42.6	41
Month 1	60.5	68	22.8	55	24.9	55	63.3	50
Month 6	64.3	68	25.6	59	21.5	45	54.0	45
Month 7	90.7	82	35.9	77	44.5	73	106.9	68
Month 12	82.9	82	33.4	73	33.6	68	79.2	68
Month 60	107.5	86	29.8	64	30.1	73	71.4	68

^*a*^ GMC—geometric mean concentration (given for IgG and IgG2 as mg/L and for IgA and IgM as U/mL); PRC—percentage comparable to reference cohort. Healthy blood donors who have not had any pneumococcal vaccination make up the reference group. Reference values were defined as ≥43.8 mg/L for IgG (18), ≥20.5 mg/L for IgG2 (18), ≥21.0 U/ml for IgA (19), and ≥54.0 U/ml for IgM (19).

To further contextualize these findings, antibody concentrations were expressed as percentage comparable to reference cohort (PRC), calculated relative to values obtained from a healthy pneumococcal vaccine-naïve reference cohort as previously described (18, 19). At baseline, IgG and IgM levels in kidney transplant recipients corresponded to 36% and 41% of the reference values for healthy controls, respectively, while IgG2 and IgA were lower (27% each). Following vaccination, all subclasses increased substantially, reaching peak PRC values at month 7 with 82% for IgG, 77% for IgG2, 73% for IgA, and 68% for IgM. At month 60, PRC values remained stable for IgG (86%) and IgA (73%), whereas IgG2 showed a modest decline to 64%, and IgM persisted at 68% of the reference values.

### Antibody kinetics in PCV20-revaccinated patients

**Fig 6:**
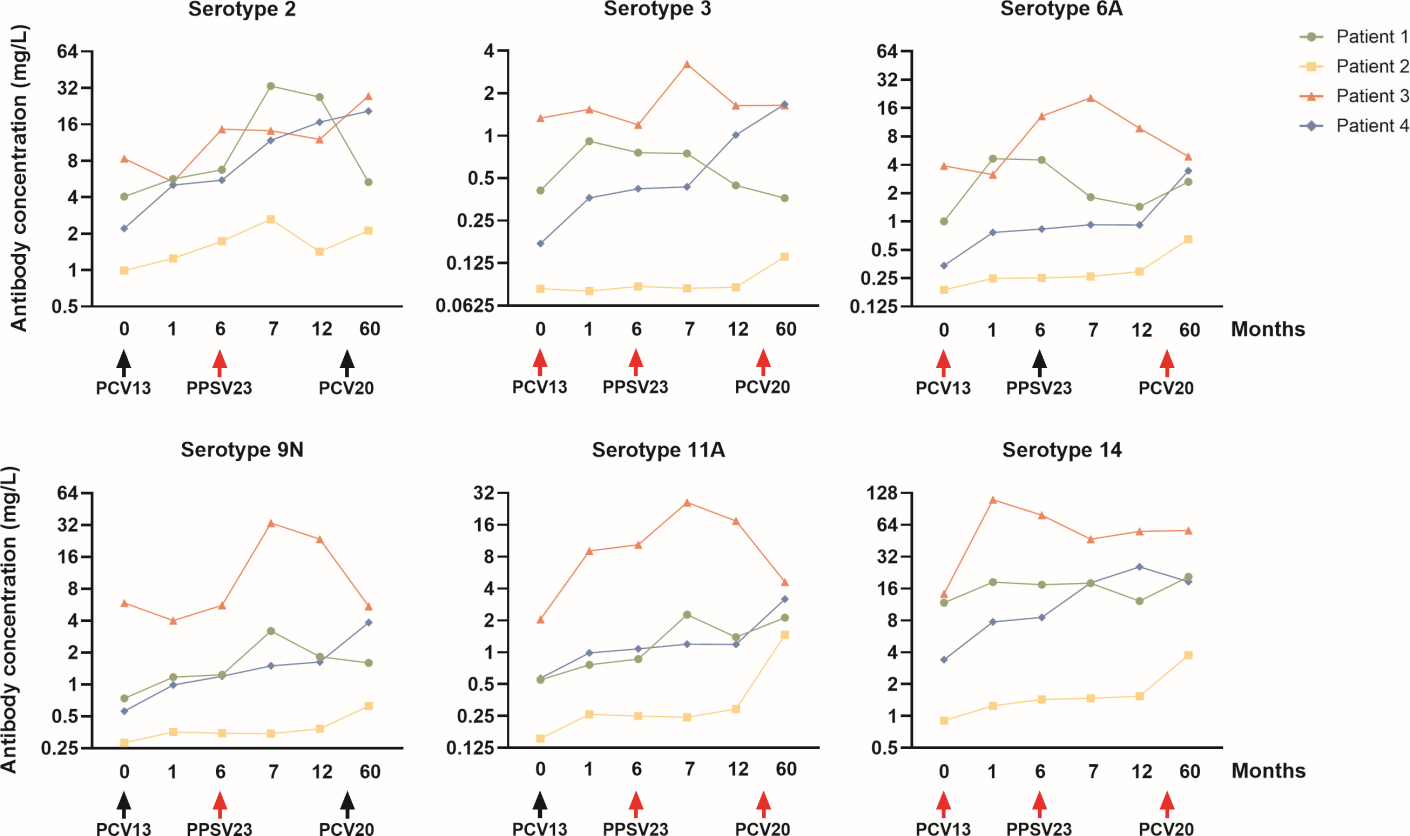
Individual course of pneumococcal antibody concentrations in the four patients who received a PCV20 booster within the last year before follow-up. Red-colored arrows indicate that the serotype is included in the respective vaccine.

Among the 26 participants re-enrolled for the 5-year follow-up, 4 patients had received an additional booster vaccination with PCV20 within the year preceding the month-60 blood collection. Figure 6 illustrates the longitudinal patterns for selected serotypes. Among the four patients, patient 1 was 83 years old, and the remaining three patients were between 41 and 54 years of age. Due to the small number of patients, no conclusions can be drawn regarding the impact of age or type of immunosuppressive therapy on serotype-specific antibody responses.

Although serotypes 2 and 9N are not included in the PCV20 formulation, minor fluctuations in antibody concentrations were observed in these serotypes, suggesting possible cross-reactive or bystander effects following booster vaccination. Responses to serotype 3 remained generally weak across most participants, consistent with its known poor immunogenicity. However, patient 4 demonstrated an exception, achieving a further increase in antibody concentration after PCV20 administration, corresponding to a 9.7-fold rise compared with baseline and surpassing the protective threshold of 1.3 mg/L with a final concentration of 1.7 mg/L. For serotype 14, which had already elicited robust antibody levels after the initial sequential vaccination, antibody concentrations remained largely stable in most patients, reflecting durable protection without additional boosting from PCV20. An exception was patient 2, who consistently showed the lowest antibody levels across serotype testing but demonstrated a further increase from 1.5 mg/L at month 12 to 3.8 mg/L at month 60, suggesting some booster-related improvement even in an initially weak response. The most pronounced post-booster increases were observed for serotypes 6A and 11A, both of which are contained in PCV20. All patients except patient 3, who already exhibited the highest antibody concentrations throughout the observation period, showed clear rises in antibody levels for these two serotypes after revaccination.

## DISCUSSION

In clinical practice, the question often arises as to when the optimal time for revaccination or for administration of one of the newer pneumococcal vaccines may be. This is particularly relevant for KTRs, in whom the immune response to vaccination is known to be reduced and to decline more rapidly than in the general population. To address this issue, we conducted a 5-year follow-up of our previously vaccinated cohort and reassessed both serotype-specific and global pneumococcal antibody concentrations after sequential immunization with PCV13 and PPSV23.

In our study, 11 out of 46 patients (23.9%) died within 5 years post-vaccination. Overall, the mortality observed in our cohort aligns with previously reported rates for kidney transplant recipients (20), where infections and cardiovascular events represent the most frequent causes of death (21). Studies analyzing large transplant populations have shown that 15–17% of deaths are infection-related, with pneumonia being a major contributor (21, 22). Of the 11 patients who died during the 5-year follow-up, the cause of death was known for only four individuals. Among these, one patient represents the only case in the cohort with a probable pneumococcal infection. Although blood cultures did not yield the pathogen, a positive urinary antigen test strongly suggested pneumococcal disease. Overall, this may be regarded as a favorable outcome over such a long observation period, with a comparatively low number of infection-related events. Particularly encouraging is the fact that none of the 26 re-enrolled participants reported any episodes of pneumonia during the 5 years following vaccination.

A recurring challenge in studies investigating pneumococcal vaccines is the absence of a universally accepted threshold for defining protective antibody concentrations in adults, particularly among immunocompromised individuals. The correlate of protection established for the pediatric population is an antibody concentration of 0.35 mg/L, derived from three clinical trials of the 7-valent pneumococcal conjugate vaccine (PCV7) (23). This threshold, adopted by the WHO for vaccine licensure in children, does not reliably translate to adult populations (24). In adult cohorts, the protective level remains uncertain and appears to vary by serotype, age, and immune status. For example, in a study of 100 healthy “pneumococcal vaccine naïve” adults vaccinated with PPSV23, serotype-specific cut-offs were derived (17) and found to cluster around higher levels to define a meaningful response (~1.3 mg/L or greater). Therefore, in adult populations, the AAAAI defines a protective or adequate response to each pneumococcal serotype as an antibody concentration equal to or greater than 1.3 mg/L (16). Moreover, it is indicated that if this threshold is achieved for at least 70% of the serotypes tested, the overall immune response is considered adequate following polysaccharide vaccination (25). However, these criteria still require validation across different immunocompromised populations, including KTRs. In a large seroepidemiological study conducted in China, more than half of healthy adults had pre-vaccination IgG concentrations ≥1.3 mg/L for several pneumococcal serotypes, yet their post-vaccination fold increases were relatively modest (26). These results indicate that individuals with high baseline antibody levels may exhibit smaller relative increases after vaccination, suggesting that absolute concentration thresholds alone are insufficient to assess vaccine responsiveness. Recently suggested serotype-specific values, approximated from healthy adults following PPSV23, can support a more nuanced assessment of vaccine-induced immunity, although they are not established or widely recognized (17). When applying these thresholds to our kidney transplant recipient cohort, it becomes apparent that for all serotypes except 2 and 14, fewer than half of the patients reached the defined protective levels, highlighting the relatively limited vaccine response in this immunocompromised population. No assessment can be made for serotype 6A, as no reference value is available.

In our study, serotype-specific differences were consistent with previous reports demonstrating that individual serotypes differ in their immunogenicity and durability of antibody response. In our cohort, antibody concentrations for serotype 14 initially increased markedly after vaccination and remained 2.4-fold higher than baseline levels at the 5-year follow-up. This aligns with prior findings in elderly individuals, where serotype 14 demonstrated the strongest persistence among all tested serotypes: the GMC increased 7.7-fold 1 month after vaccination and remained 3.0-fold higher 3 years later compared with pre-vaccination levels (8).

Serotypes 3, 9N, and 11A have long been included in commonly used pneumococcal vaccines, yet they continue to represent predominant serotypes causing pneumococcal infections and isolates across Europe (27, 28). When applying a serotype-specific response threshold (absolute concentration ≥1.3 mg/L and a ≥2-fold increase from baseline) as a criterion for adequate vaccine response, serotype 3 showed an overall below-average performance in both the short-term and long-term analysis in our cohort. After 12 months, only about half of the recipients achieved at least a twofold increase in antibodies against serotype 3 compared with baseline values. In the long-term follow-up, merely 14% of patients showed antibody concentrations above the general protective threshold of 1.3 mg/L, and likewise above the serotype-specific cut-off of 1.8 mg/L. This observation is consistent with previous findings showing that serotype 3 induces a relatively weak immune response even when included in conjugate vaccines, and that antibody concentrations against serotypes 3 and 6B are significantly lower in elderly individuals compared with younger adults (29). Experimental data indicate that the release of capsular polysaccharides (CPS) from *Streptococcus pneumoniae* type 3 interferes with antibody-mediated killing and protection by anti-CPS antibodies, which may explain the limited vaccine efficacy observed for this serotype (30). In our study, four patients received a PCV20 booster during follow-up. Two of these patients showed an increase in serotype 3 antibody levels compared with month 12, while two maintained stable concentrations ≥0.35 mg/L. In the remaining 22 participants who did not receive a booster, antibody levels also remained stable. These results suggest that a PCV20 booster may temporarily enhance serotype 3 responses and may provide some short-term benefit. However, given the limited effectiveness of existing vaccination strategies in immunocompetent individuals and the continued dominance of serotype 3 in invasive pneumococcal disease, a booster vaccination alone is unlikely to resolve the ongoing issue (4).

Although the GMCs in our cohort remained largely stable between month 12 and the 5-year follow-up, the serotype-specific antibody levels were generally lower compared with those reported for healthy adults at similar time points. In KTRs, corticosteroids, mycophenolic acids, and calcineurin inhibitors exert broad immunosuppressive effects, particularly targeting T-cell activation and proliferation (31). Since conjugate pneumococcal vaccines such as PCV13 rely on antigen-specific T-cell help to stimulate memory B cells and promote the proliferation of antibody-secreting plasma cells, their effectiveness can be substantially impaired under such immunosuppressive regimens (32). Consequently, KTRs often exhibit a reduced humoral response compared with healthy adults (33). In our cohort, immediately following sequential vaccination with PCV13 and PPSV23, antibody concentrations increased markedly; however, not all patients achieved total antibody levels comparable to those typically observed in healthy individuals (34). This heterogeneity underscores the impact of immunosuppressive therapy on vaccine-induced immunity. From a clinical perspective, it is, however, encouraging that none of the re-enrolled participants developed pneumonia or IPD during the observation period. Despite the comparatively lower antibody concentrations, this finding suggests that the attenuated immune response observed in KTRs may still provide adequate protection.

Because plain polysaccharide vaccines are generally not thought to induce long-lasting immunological memory, and since the risk of pneumococcal infection increases with advancing age and ongoing immunosuppression, revaccination may help sustain protective antibody levels and further reduce the likelihood of invasive pneumococcal disease.

In terms of the global immunoglobulin values, we had expected a decline following the sequential vaccination series; indeed, a modest drop was seen for IgM, IgA, and IgG2, but by no means as pronounced as anticipated. In fact, IgG even showed a slight increase, driven most likely by three outlier patients with exceptionally high concentrations. One plausible explanation for these outliers is subclinical exposure to pneumococci, which may have transiently boosted antibody levels without causing overt infection. Humoral immunity against the pneumococcal proteome is acquired through multiple episodes of pneumococcal exposure, including asymptomatic carriage or subclinical infections (35). In adults, repeated exposure can lead to variable antibody responses, potentially explaining some of the observed heterogeneity in our cohort (35, 36). Building on these observations, it is worth considering that local immune responses at the mucosal surface could provide additional protection. Although our study focused on serum IgA, which may not fully capture mucosal immunity, mucosal delivery of a pneumococcal booster vaccine could theoretically induce stronger local IgA responses in the respiratory tract, the primary site of pneumococcal entry, thereby complementing systemic antibody-mediated protection (37).

Given the known waning over time or general low antibody levels and the increasing availability of broader-spectrum conjugate vaccines, revaccination strategies such as PCV20 boosting have gained growing interest as a potential approach to restore or enhance protection. In our cohort, four of the original 46 participants received a booster vaccination with PCV20 within the year preceding the follow-up blood sampling at 60 months. According to the current recommendations of the Centers for Disease Control and Prevention (CDC), individuals with immunocompromising conditions who have previously completed a sequential pneumococcal vaccination series should receive one dose of PCV20 or PCV21 5 years after their most recent pneumococcal vaccine (38). However, in the case of our four patients, the booster vaccination was administered somewhat earlier than recommended based on the CDC guidelines. Nevertheless, our analysis indicates several positive effects associated with this revaccination. Most notably, irrespective of the interval since the previous vaccination, the use of PCV20 provides coverage against additional serotypes that were not included in the earlier vaccine formulations, thereby broadening the overall protective spectrum for these patients.

Among the four patients receiving a PCV20 booster, one was on belatacept therapy. In the broader cohort of belatacept-treated patients, no significant differences in IgG responses were observed. The belatacept-treated patient receiving the PCV20 booster (patient 3) exhibited generally higher antibody concentrations compared with the other three booster recipients; however, this patient did not show a pronounced increase in response to the booster itself, possibly due to the effects of belatacept on T cell-dependent antibody induction.

The age range within our kidney transplant cohort was broad, reflecting the heterogeneity of underlying diseases leading to transplantation. Aging is known to impair vaccine responses through immunosenescence, including reduced B-cell diversity and reduced production of naïve T cells (39). In studies of healthy older adults following PPSV23 vaccination, serotype-specific IgG concentrations measured one month post-vaccination were generally reported at relatively high levels. For example, in some cohorts of older healthy vaccinees, serotype 14 reached a GMC of approximately 15.5 mg/L (40). In additional data sets, serotype-specific responses among elderly subjects have been described with GMCs of 10.1 mg/L for serotype 2, 3.3 mg/L for serotype 3, 16.0 mg/L for serotype 9N, and 19.2 mg/L for serotype 14 (41). In contrast, in our kidney transplant recipient cohort, corresponding GMCs after sequential PCV13 and PPSV23 vaccination were substantially lower, underscoring the pronounced impact of immunosuppression on vaccine-induced antibody responses.

Concerns regarding the safety of pneumococcal revaccination have historically influenced guidance on timing. Early reports from the 1970s and 1980s suggested that revaccination of healthy children and adults within a few years of the initial dose could lead to an increased frequency and severity of local injection-site reactions (42–44). However, more recent studies in older adults have consistently shown that a second pneumococcal vaccination administered 5 or more years after the first is well tolerated (45–47). In fact, a prospective trial of 1,414 adults aged 50–74 years found that revaccination with PPSV23, even among those previously vaccinated, caused only mild and self-limited local reactions (48). A phase III trial in 1,991 healthy infants demonstrated that a four-dose series of PCV20 was well tolerated and elicited robust serotype-specific immune responses, with a safety profile comparable to PCV13 and no serious adverse events (49). To date, no major differences in adverse events have been observed between children and adults over many years of pneumococcal vaccination, indicating a consistent safety profile across age groups. Especially in KTRs, multiple studies have shown that vaccination against *S. pneumoniae* does not induce antibodies against HLA or MICA in clinically stable patients, suggesting that pneumococcal vaccines are immunologically safe for KTRs (50, 51). Even in the four patients who were additionally vaccinated during the follow-up (albeit a very small cohort), PCV20 vaccination was safe in the short term, with no occurrences of allograft rejection or development of donor-specific antibodies (DSAs).

This study has several limitations that should be acknowledged. First, the number of patients available for long-term follow-up was relatively small, limiting statistical power and the ability to perform robust subgroup analyses. Second, the cohort exhibited a considerable age variation, which may influence immune responsiveness and antibody persistence. Although exploratory age-stratified analyses were conducted, no meaningful differences were observed, likely due to the limited sample size. Third, only patients who survived and consented to re-enrollment were available at the 5-year time point, introducing a potential selection bias toward clinically stable individuals.

To conclude, antibody concentrations remained relatively stable over 5 years following sequential vaccination, although overall concentrations were lower than in healthy individuals. Importantly, during this follow-up period, no cases of invasive pneumococcal disease or bacterial pneumonia were observed in the re-enrolled cohort. Taken together, these findings support the safety and clinically observed protection of revaccination independent of antibody levels. In line with current international recommendations, efforts should focus on improving vaccine coverage in high-risk populations under immunosuppression. Larger studies are needed to further define efficacy and safety, but conducting such trials remains challenging.

## Data Availability

The data presented in this study are available on request from the corresponding author.
